# Canine myxosarcomas, a retrospective analysis of 32 dogs (2003–2018)

**DOI:** 10.1186/s12917-019-1956-z

**Published:** 2019-06-27

**Authors:** Yoshimi Iwaki, Stephanie Lindley, Annette Smith, Kaitlin M. Curran, Jayme Looper

**Affiliations:** 1 The Department of Clinical Science, College of Veterinary Medicine, Auburn University, 1220 Wire Road, Auburn, AL 36849 USA; 2 Department of Clinical Sciences, Carlson College of Veterinary Medicine, Oregon State University, 172 Magruder Hall, Corvallis, OR 97331 USA; 3 Department of Veterinary Clinical Sciences, School of Veterinary Medicine, Louisiana State University, Skip Bertman Dr, Baton Rouge, LA 70803 USA

**Keywords:** Dog, Myxosarcoma, Soft tissue sarcoma

## Abstract

**Background:**

Myxosarcomas are known to be classified as soft tissue sarcomas. However, there is limited clinical characterization pertaining specifically to canine cutaneous myxosarcomas in the literature. The objective of this study is to evaluate the local recurrence rate, metastatic rate and prognosis of canine myxosarcoma.

**Results:**

A total of 32 dogs diagnosed with myxosarcoma via histopathology were included in this retrospective study. All dogs had surgical resection. No adjunct treatments were performed in 9 dogs, while 22 dogs also received either radiation therapy or chemotherapy, or a combination of both. One dog received only NSAID after surgery. Overall median survival time (MST) was 730 days (range 20–2345 days). The MST of dogs with a tumor mitotic count < 10/10 HPF was 1393 days (range 20–2345 days). The dogs with a tumor mitotic count of 10 or greater/10 HPF had a MST of 433 days (range 169–831 days). There was no significant difference of MST among different treatment modalities. Local recurrence was noted in 13 cases (40.6%) and the median time to recurrence was 115.5 days (range 50–1610 days). The median time to local recurrence in dogs with mitotic count of < 10/10 HPF was 339 days (range 68–1610 days) and in dogs with mitotic count of 10 or greater/10 HPF was 119 days (range 50–378). Metastasis to local lymph node or lung was noted in 8 cases (25%) with median time to metastasis of 158.5 days (range 0–643 days).

**Conclusions:**

Based on the results of this retrospective study, myxosarcoma may have a higher local recurrence rate and risk of metastasis to the local lymph nodes compared to other soft tissue sarcomas.

## Background

Soft tissue sarcomas (STSs) are a heterogeneous group of tumors that arise from mesenchymal tissues, including muscle, adipose, neurovascular, fascial, and fibrous tissue. They account for 15% of all skin and subcutaneous tumors in the dog [1]. Most STSs are solitary tumors in middle-aged to older dogs. There is no specific breed or sex predilection. Malignant neoplasms in this category include fibrosarcoma, peripheral nerve sheath tumor, myxosarcoma, undifferentiated sarcoma, liposarcoma, malignant fibrous histiocytoma, and rhabdomyosarcoma [2, 3]. STSs generally appear as pseudoencapsulated tumors but have poorly defined histologic margins or infiltrate through and along fascial planes. Histopathologic grade, along with margins, predicts metastasis and local recurrence. Low grade STSs have a less than 15% metastatic rate and grade III STSs have a reported 41% metastatic rate [4]. With incomplete resection, the local recurrence rate of grade I STS is 7%, grade II is 34%, and grade III is 75% [5].

Myxosarcomas are classified as soft tissue sarcomas. This neoplasm is of fibroblast origin with an abundant myxoid matrix composed of mucopolysaccharides [6]. The most commonly reported locations for myxosarcomas are the trunk and limbs in the dog [6]. They have also been reported in the spleen, heart, eye, brain, spine, vertebra, lung and temporomandibular joint [7–22]. There has been limited clinical characterization of canine cutaneous myxosarcomas specifically in the literature.

In humans myxofibrosarcoma represents about 5% of all soft tissue sarcomas. It affects older patients with the most common sites of presentation being the extremities (77%), followed by trunk (12%) and head and neck region (3%) [23]. Myxofibrosarcomas are locally aggressive tumors that have a propensity for local recurrence. The local recurrence rates range from 16 to 57% and 25–52% of patients develop multiple recurrences [24, 25]. Margin status and tumor grade have been evaluated in relationship to local recurrence: no local recurrence was noted with a margin of at least 1 cm, whereas 40% of patients had local recurrence with a less than 1 cm margin [26]. The risk of local recurrence ranged from approximately 48% for low-grade tumors to 62% for high-grade tumors [27]. Overall 5-year metastasis-free survival is reported as 47 to 90% [24, 25, 28]. Currently, wide surgical resection with 2 cm soft tissue margins is the mainstay of treatment in humans.

To the authors’ knowledge, no large data set of canine myxosarcoma has been published. The purpose of this study was to evaluate the local recurrence rate, metastatic rate and prognosis of canine myxosarcomas.

## Results

### Patient characteristics

Thirty two dogs were included in this study. Median age was 10 years (range 3.5–14 years) and median body weight was 25.0 kg (range 5.8–58 kg). Gender distribution included 20 spayed females and 12 castrated males. Breeds included Labrador retriever (*n* = 10), mixed breed dog (*n* = 6), Australian shepherd (*n* = 3), beagle (*n* = 2), golden retriever (*n* = 2), miniature dachshund (*n* = 2), and one each of border collie, English bull dog, German shorthair pointer, mastiff, Staffordshire terrier and toy poodle. Tumors were located on the head for 7 dogs, trunk for 18 dogs, and limbs for 7 dogs (thoracic limb in 2 dogs and pelvic limb in 5 dogs). The tumor size was available in 20 dogs and the median size of the longest axis was 7.45 cm (range 1–31 cm). The duration of clinical signs, which was available for 28 dogs, ranged from 1 week to 4 years with a median of 1.5 months.

### Initial staging

Hematological and serum biochemical analysis were available in 30 dogs. Common abnormal clinicopathologic parameters included mild increased liver enzymes (*n* = 7). Mildly elevated blood urea nitrogen was noted in 1 dog and mild anemia was noted in 1 dog. Thoracic radiographs were performed in 24 cases and thoracic CT scans were performed in 5 cases. No pulmonary metastasis was noted in any dogs with thoracic imaging. Abdominal ultrasound was performed in 10 dogs. The abnormal findings included enlarged adrenal glands (*n* = 3), hyper- or hypo-echoic liver nodules (*n* = 2), splenic nodule (*n* = 2), renal cysts (*n* = 2), enlarged medial iliac lymph nodes (*n* = 2), and bilateral nephrocalcinosis (*n* = 1). Fine needle aspirations of enlarged medial iliac lymph nodes were performed, however, the samples were non-diagnostic in both dogs.

### Histopathology

All dogs received surgery. The median number of surgeries was 1 (range 1–5). A complete margin was achieved in 12 dogs, and 15 dogs had incomplete margins. Margin status was not reported in 5 dogs. Grades were available in 11 cases; of these, 4 tumors were classified as grade I, 2 tumors as grade II, 1 tumor as grade III, and 4 tumors as low grade. Mitotic counts were available in 29 cases; of these, 20 tumors had a mitotic count of less than 10/10 high-power fields (HPF), 3 tumors had 10–19/10 HPF, and 6 tumors had more than 19/10 HPF. There was no correlation between margin status and mitotic count (Table 1).

**Table 1:** Margin status when classified with mitotic count

Mitotic count (/10HPF)	Margin complete	Margin incomplete
< 10	8 dogs	9 dogs
10–19	1 dog	2 dogs
> 19	3 dogs	3 dogs

### Adjuvant therapy

Nine dogs did not receive any treatment after surgery. Pre-operative radiation therapy was performed in one dog and intra-operative radiation therapy was done in one dog. Five dogs received post-operative radiation therapy, 8 dogs received post-operative chemotherapy, and 7 dogs received a combination of both. One dog received only a non-steroidal anti-inflammatory drug following surgery. Margin status for each group were summarized in Table 2. Fourteen dogs were treated with radiation therapy and the total number of courses of radiation therapy was 18 courses. Among the 14 dogs that received radiation therapy, definitive radiation therapy was performed in 9 dogs, hypo-fractionated radiation therapy in 8 dogs and intra-operative radiation therapy in 1 dog. Four dogs received second courses of radiation therapy after local recurrence. The intervals between the completion of first radiation therapy and the initiation of second radiation therapy were 45 days and 55 days for two dogs who received hypo-fractionated radiation therapy, and 342 days and 1736 days for two dogs who received definitive radiation therapy. The total dose of definitive radiation therapy ranged from 50 to 63 Gy given in 2.5- to 3-Gy daily fractions on a Monday through Friday schedule (the median total dose was 57 Gy). Two dogs could not complete definitive radiation therapy due to side effects; one dog received a total of 24 Gy in 8 treatments and discontinued due to grade 2 moist desquamation, the other dog received a total of 36 Gy in 12 treatments and discontinued due to mucositis with unknown grade. The total dose of hypo-fractionated radiation therapy ranged from 15 to 32 Gy (the median total dose of 20 Gy). Hypo-fractionated radiation was delivered daily fractions for 5 days in a row, biweekly fractions or once a week fractions. The dog treated with intra-operative radiation therapy received 14 Gy. The chemotherapy drugs which were used included metronomic chemotherapy (*n* = 11; cyclophosphamide was used in 11 dogs, chlorambucil was used in 2 dogs, melphalan was used in 1 dog), doxorubicin (*n* = 6), Palladia (*n* = 4), Mastinib (*n* = 2), Rapamycin (*n* = 1), and CCNU (*n* = 1). The median dose of cyclophosphamide was 15 mg/m^2^ once a day (range 8.4 to 25 mg/m^2^). Chlorambucil was given 0.1 mg/kg once a day, and 0.15 mg/kg once a day for each dog. Melphalan was given 0.076 mg/kg once a day. Doxorubicin was given 30 mg/m2 (> 10 kg dogs) or 1 mg/kg (< 10 kg dogs) once every 3 weeks. The median dose of Palladia was 2.4 mg/kg on Monday, Wednesday, and Friday (range 2.3 to 2.6 mg/kg). Mastinib was given 9 mg/kg every other day. Rapamycin was given 0.1 mg/kg on Monday, Wednesday, and Friday. CCNU was given 60 mg/m2 once every 3 weeks. Seven dogs received multiple chemotherapy drugs due to disease progression. The relationship between mitotic count and treatments are summarized in Table 3.

**Table 2:** Margin status of each treatment groups

Treatment	Margin complete	Margin incomplete	Unknown for margin status
No treatment	4 dogs	3 dogs	2 dogs
Pre-operative radiation therapy		1 dog	
Intra-operative radiation therapy	1 dog		
Post-operative radiation therapy alone		3 dogs	2 dogs
Post-operative chemotherapy	5 dogs	3 dogs	
Post-operative radiation therapy and chemotherapy	2 dogs	4 dogs	1 dog
NSAID alone		1 dog

**Table 3:** The correlation between mitotic count and treatments which performed

Mitotic count (/10HPF)	Treatments
< 10	No treatments (*n* = 8)
	NSAIDs alone (*n* = 1)
	Radiation therapy alone (*n* = 4)
	Radiation therapy + chemotherapy (*n* = 2)
	Chemotherapy alone (*n* = 5)
10–19	Radiation therapy + chemotherapy (*n* = 3)
> 19	Radiation therapy alone (*n* = 1)
	Radiation therapy + chemotherapy (*n* = 2)
	Chemotherapy alone (*n* = 3)

### Outcomes

Fifteen cases were censored; 8 dogs were lost to follow-up (median time of follow-up was 587 days; range 77–1825 days). Of these 8 dogs, 3 dogs had progressive disease at the time of last contact. This included metastasis to medial iliac lymph node at day 77, local recurrence and metastasis to sternal lymph node, aortic lymph node, lungs, and subcutaneous tissue at day 170, and local recurrence at day 566. Seven dogs were still alive at the time of data collection (median time of last follow up 287 days; range 158–694 days).

**Fig. 1:**
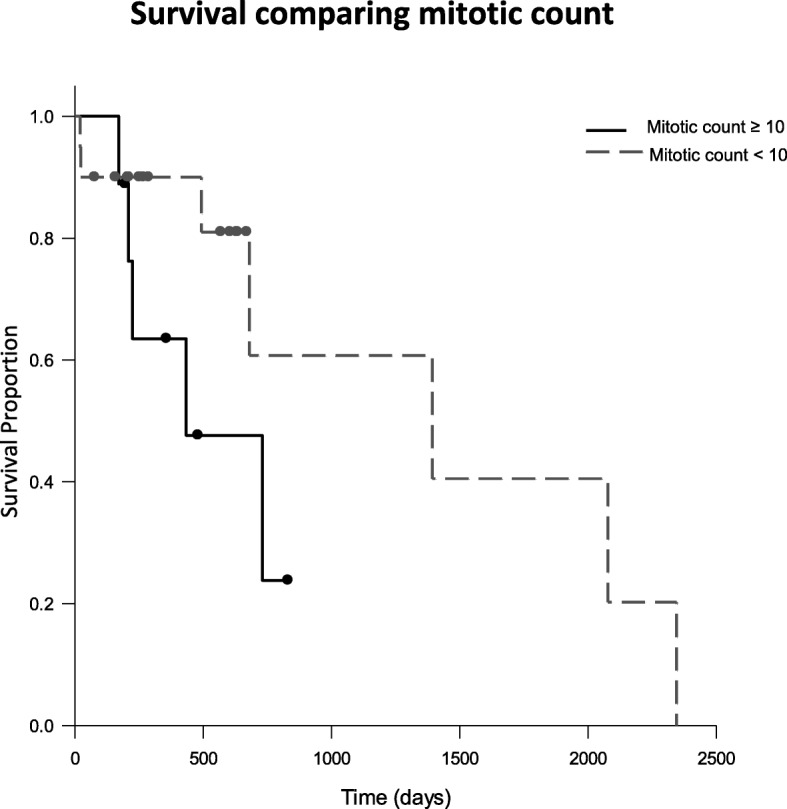
Kaplan-Meier survival curves for dogs with tumor mitotic count < 10/10 HPF (dashed line) and mitotic count ≥10/10 HPF (solid line). The MST for dogs with tumor mitotic count < 10/10 HPF was 1393 days and dogs with tumor mitotic count ≥10/10 HPF was 433 days. (*P* = 1.019)

**Fig. 2:**
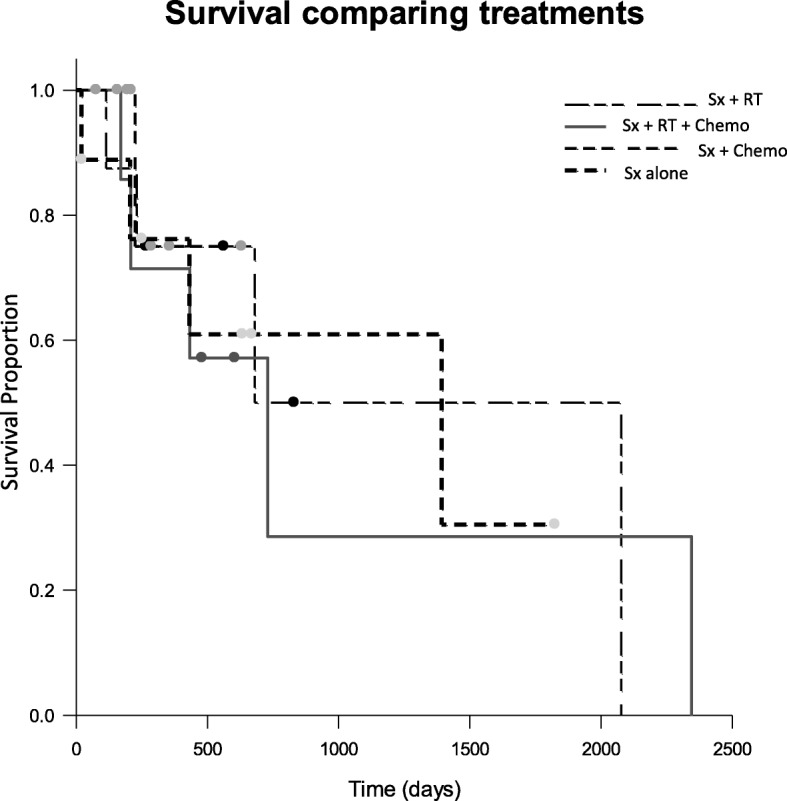
Kaplan-Meier survival curves for dogs treated with no adjuvant therapy (heavy dashed line), surgery and chemotherapy (dashed line), surgery, radiation therapy and chemotherapy (solid line), and surgery and radiation therapy (Unequal dashed line). The MST for dogs treated with surgery alone, surgery, radiation therapy and chemotherapy, and surgery and radiation were 1393 days (range 20–1805 days), 730 days (170–2345 days), and 680 days (231–2077 days), respectively. More than a half of dogs treated with surgery and chemotherapy were censored and the MST was not calculated

Overall MST was 730 days (range 20–2345 days). The MST of dogs with a tumor mitotic count < 10/10 HPF was 1393 days (range 20–2345 days). The dogs with a tumor mitotic count of 10 or greater/10 HPF had a MST of 433 days (range 169–831 days) (Fig. 1). There was no significant difference (*p* = 0.109) between these two median survival times. When comparing the use of adjuvant therapy, no significant difference was noted in the MST between dogs receiving no adjuvant therapy, chemotherapy alone, radiation therapy alone, or a combination of chemotherapy and radiation therapy (Fig. 2). There was no significant difference between complete excision and incomplete excision in the dogs who only had surgery as a treatment. Local recurrence was noted in 13 cases (40.6%). Local recurrence was confirmed by cytology in 6 cases, histopathology in 2 cases, CT scan in 1 case, and method was not reported in 4 cases. The median time to local recurrence was 115.5 days (range 50–1610 days). The tumor recurred in four of the 20 dogs with tumors that had a mitotic count of < 10/10 HPF (20%), in all dogs with tumors with a mitotic count of 10–19/10 HPF (100%), and in four of the 6 dogs with a mitotic count of > 19/10 HPF (66.7%). The median time to local recurrence in dogs with mitotic count of < 10/10 HPF was 339 days (range 68–1610 days) and in dogs with mitotic count of 10 or greater/10 HPF was 119 days (range 50–378). Metastasis was noted in 8 cases (25%). Metastasis was confirmed by cytology in 1 dog and by histopathology in 1 dog. In 5 dogs, metastasis was presumed by imaging tests (CT scan, thoracic radiographs, and abdominal ultrasound). Method was not reported in 1 dog. The metastatic locations were regional lymph nodes in 5 dogs and distant organs, including lungs, distant lymph node, and subcutaneous tissue, in 4 dogs. The median time to metastasis was 158.5 days (range 0–643 days). Metastasis was noted in three of the 20 dogs with a mitotic count of < 10/10 HPF (15%), in two of the 3 dogs with a mitotic count of 10–19/10 HPF (66.7%), and in three of the 6 dogs with a mitotic count of > 19/10 HPF (50%).

## Discussion

In general soft tissue sarcomas are a broad category of multiple different histologic tumor subtypes that are often not separately evaluated in the literature. In this study 32 cases of histologically diagnosed myxosarcoma were retrospectively collected at five institutions. Results of this study may suggest the clinical behavior of myxosarcoma is different from other types of soft tissue sarcomas.

In this study no statistically significant difference was found between survival times based on mitotic count. Mitotic count for soft tissue sarcomas as a whole has previously been reported as predictive for survival time with MST of 1444 days for those with MI <  10/10 HPF, and 236 days with MI >  19/10 HPF [4]. There were only 29 cases of myxosarcoma in this study for which mitotic counts were available, further decreasing the number of overall cases in each sub-category of mitotic count. This small number of cases could be the reason for no statistical difference found despite the 960 day numeric difference in MST between patients with tumors having a mitotic count < 10/10 HPF or ≥ 10/10 HPF. Treatment bias may also represent a possible reason for no significant difference between mitotic count and prognosis in this study. Sixty-seven percent of dogs with tumor having a lower mitotic count received no adjuvant therapy after surgery, while all dogs with tumors having a mitotic count of 10/10 HPF or higher received some type of adjuvant therapy. Larger prospective studies are needed to ascertain the true significance of mitotic count as a predictor of survival for canine myxosarcoma.

Local recurrence was noted in 13 cases (40.6%) with the median time to recurrence of 115.5 days in this study. Two of these cases had complete surgical margins as assessed on histopathology. In the current study, 20% of myxosarcoma with mitotic count < 10/10 HPF recurred and 77.8% of the tumors with mitotic count ≥10/10 HPF had local recurrence. Reported median time to local recurrence ranges from approximately 6 months to 26 months when soft tissue sarcomas are treated with surgery, or combination of surgery, radiation therapy, and chemotherapy [5, 29–32]. It is difficult to compare this study with the previous reports; however, even with multimodal treatments, the local recurrence rate of myxosarcoma appears to be higher with median time to local recurrence shorter than previously reported soft tissue sarcoma as a whole.

Eight dogs in this study developed metastatic disease with an overall metastatic rate of 25%. This study is a multi-institutional study and not all the patients were regularly screened for disease progression; thus this metastatic rate could be higher. In a previous study of 75 dogs with soft tissue sarcomas treated with surgery alone, the reported overall metastatic rate was 17% [4]. While the overall metastatic rate of myxosarcoma compared to soft tissue sarcomas in general does not differ greatly, of interest noted in this study, 6 dogs (18.7%) had lymph node metastasis. Previously reported the evidence of lymph node metastasis for soft tissue sarcomas has only been 6% [4]. This finding may suggest myxosarcoma to have a higher metastatic rate to the local lymph nodes. For soft tissue sarcomas treated with surgery alone the previously reported median time to metastasis was 365 days (range 0–1444 days), while 39 dogs treated with definitive radiation therapy and doxorubicin after incomplete resection demonstrated a metastatic rate of 15.3% with the median time to metastasis of 276 days (range 8–826 days) [4, 33]. In our study the median time to metastasis was 158.5 days, despite 20 patients receiving some form of adjuvant treatment following surgery. Therefore, the metastatic interval may be shorter for myxosarcoma than for other soft tissue sarcomas.

No significant difference was noted between survival times of dogs when separated out into treatment groups. The reason for this may be inherent clinician bias for treatment plans with no adjuvant treatment chosen mostly for dogs with lower mitotic count tumors and complete surgical margins. On the other hand, dogs with higher mitotic count tumors and/or incomplete surgical margins tended to have adjuvant treatments after the surgery.

The limitations of the current study are inherent to its retrospective nature and small sample size. The initial staging tests were not completed in all cases. Histopathology reports for initial surgical procedures and previous treatment information before referral to a specialty hospital were missing for some cases. Necropsy data was available only one patient. Additionally this is a multi-institutional study; thus different pathologists read the histopathology, and treatment protocols varied and were dependent on multiple clinicians’ preferences.

## Conclusions

Myxosarcoma has been classified into the broad category of soft tissue sarcomas, and its clinical behavior in the past has not been separately evaluated. This study indicates that myxosarcoma may have a higher local recurrence rate and shorter time to recurrence even with adjuvant treatments. Additionally, the risk of metastasis to lymph nodes appears to be higher as compared to previous reports for soft tissue sarcomas as a whole.

## Methods

The medical records of dogs diagnosed with histologically confirmed myxosarcoma at five institutions (Oregon State University Lois Bates Acheson Veterinary Teaching Hospital, Auburn University Wilford & Kate Bailey Small Animal Teaching Hospital, Louisiana State University Veterinary Teaching Hospital, Hope Veterinary Specialists, Marqueen Pet Emergency & Specialty, and Orchard Park Veterinary Medical Center) between June 2003 and January 2018 were retrospectively reviewed. The following data were retrieved from medical records: age, sex, breed, body weight at the first visit, blood work results, results of diagnostic staging tests, location and size of tumor, date of surgery, histopathology results, treatments after surgery, follow-up staging tests, date of local recurrence and metastasis, date and cause of death or euthanasia, and availability of necropsy findings. Anemia was graded by hematocrit (mild as 30–36%, moderate as 18–29%, severe as < 18%). The elevation of liver enzyme was graded as mild if it was less than 5 times of upper reference range, moderate if it was 5–10 times of upper reference range, and markedly if it was more than 10 times of upper reference range.

### Statistics

Overall survival time was defined as the time from the date of diagnosis to the date of death/euthanasia. Dogs that were still alive or lost to follow-up at the time of the analysis were censored from the overall survival analysis. The time to local recurrence was defined as the time from the date of surgery to the date of recurrence. Metastasis free interval was defined as the interval between surgery and evidence of metastasis. The Kaplan-Meier method was used to calculate median survival time. The Log rank test was used to investigate survival distribution between dogs with lower- and higher-mitotic count and dogs with different treatments. A *P* value of < 0.05 was considered to be statistically significant. Commercially available software was used for all statistical calculations.^1^

## Data Availability

Data available on request from the authors.

## Abbreviations

HPF: High power field
MST: Median survival time
STS: Soft tissue sarcoma

## Footnotes

1 SigmaPlot for Windows Version 12.0, 2011, Systat software, Inc. San Jose, California
